# Relapse Rate and Associated-Factor of Recurrence after Stopping NUCs Therapy with Different Prolonged Consolidation Therapy in HBeAg Positive CHB Patients

**DOI:** 10.1371/journal.pone.0068568

**Published:** 2013-07-03

**Authors:** Xingfei Pan, Ka Zhang, Xiaoan Yang, Jiayi Liang, Haixia Sun, Xuejun Li, Yong Zou, Qingqiang Xu, Geng An, Gang Li, Qihuan Xu

**Affiliations:** 1 Department of Infectious Diseases, The 3rd Affiliated Hospital, Sun Yat-Sen University, Guangzhou, China; 2 Ultrasound Division, The 3rd Affiliated Hospital, Sun Yat-Sen University, Guangzhou, China; 3 Department of Blood Transfusion, The 3rd Affiliated Hospital, Sun Yat-Sen University, Guangzhou, China; 4 Department of Dermatology, Zhengzhou Children’s Hospital, Zhengzhou, China; 5 Department of Reproductive Medicine Center, Key Laboratory for Major Obstetric Diseases of Guangdong Province, The 3rd Affiliated Hospital of Guangzhou Medical University, Guangzhou, China; Karolinska Institutet, Sweden

## Abstract

**Background:**

Many chronic hepatitis B (CHB) patients recur after off-therapy and have to accept prolonged consolidation therapy with NUCs. We investigated the rate of HBV relapse after stopping NUCs therapy with different time period of prolonged consolidation therapy in HBeAg positive CHB patients, and analyzed the associated-factor of recurrence.

**Methods:**

We recruited 162 HBeAg-positive CHB patients who met the standard of stopping NUCs therapy recommended by the 2005 APASL. Patients in group A, without the prolonged consolidation therapy, were as controls. Patients in group B were divided into 3 subgroups (group B1, 7 (range 3–11) months of the prolonged consolidation therapy; group B2, 17 (range 13–20) months of the prolonged consolidation therapy; group B3, 28 (range 25–34) months of the prolonged consolidation therapy). Virologic relapse was defined as an increase in serum HBV DNA to >10^3^copies/ml after off-therapy.

**Results:**

One hundred and thirty-six patients (group A, 40 patients; group B1, 54 patients; group B2, 23 patients; group B3, 19 patients) were eligible for this study. The cumulative rates of relapse in group B at 6 months and 48 months were 29.2%, 41.7% after off-therapy, respectively. The cumulative rates of relapse in group B were statistically lower than that in group A at the same time periods. The cumulative rate of relapse in group B3 or group B2 was statistically lower than that in group B1, respectively. On multivariate analysis by Cox’s proportional hazard model, age at off-therapy, baseline ALT and the different time period of the prolonged consolidation therapy were associated with the relapse of HBV after off-therapy.

**Conclusions:**

Consolidation therapy with NUCs after HBeAg seroconversion should be further prolonged. Age at off-therapy, ALT at baseline and the time period of the prolonged consolidation therapy could provide information to direct anti-viral therapy.

## Introduction

There are approximately 350–400 million people chronically infected with hepatitis B virus (HBV) in the world [1]. Chronic hepatitis B (CHB) may evolve to cirrhosis and hepatocellular cancer (HCC). It is reported that HBV-related end stage liver disease or HCC is responsible for beyond 0.5–1 million deaths every year worldwide [2]–[4]. Hepatitis virus B (HBV), a hepatotropic double stranded DNA virus, is not directly cytopathic for hepatocyte, but the immune response to virus or viral antigens is thought to be responsible for liver damage or virus clearance in patients with acute and chronic HBV infection [5]. So it is very important to directly aim at HBV with antiviral therapy.

Nowadays, 4 kinds of nucleos(t)ide analogues (NUCs), Lamivudine (LAM), Adefovir Dipivoxil (ADV), Telbivudine (LDT), Entecavir (ETV) are used to treat chronic HBV infection in China. NUCs can effectively inhibit replication of HBV, but not eradicate HBV in hepatocyte. Many CHB patients recur after NUCs are discontinued. So CHB patients have to accept long-term therapy of NUCs. Long-term therapy brings many problems to CHB patients, such as high expenses, HBV drug resistance, etc.

Different researches recommend different end points of therapy with NUCs. According to guidelines recommended by the Asian Pacific Association for the Study of the Liver (APASL) [6]–[7], in HBeAg positive CHB patients, therapy could be stopped when HBeAg seroconversion with undetectable HBV DNA has been documented on two separate occasions at least 6 months apart. According to the 2009 AASLD guidelines [8], therapy may be discontinued in patient who has confirmed anti-HBe seroconversion (detection on 2 occasions 1–3months apart) and has completed at least 6 months of consolidation therapy after the appearance of anti-HBe. However, the rate of relapse is still high after off-therapy, even if CHB patient is treated with at least 6 months of consolidation therapy [9]. So the time period of the consideration therapy and the associated factor of recurrence should be evaluated again.

In the present study, we analyzed the relapse rate of HBV and the associated-factor of recurrence after stopping NUCs therapy with the different time period of the prolonged consolidation therapy in HBeAg positive CHB patients. These patients met the standard of stopping therapy recommended by the 2005 APASL guideline [6] and were given the different time period of the prolonged consolidation therapy.

## Materials and Methods

### Patients

Patient recruitment started in January 2001 and the last patient follow-up was in March 2012. We recruited 162 HBeAg-positive CHB patients who were referred to Department of Infectious Diseases, the Third Affiliated Hospital of Sun Yat-sen University, Guangzhou, China. These patients met the standard of stopping NUCs therapy recommended by the 2005 APASL. The standards for diagnosis of CHB have been previously described in detail [10]. The included standards were as follows: (1) HBsAg positive, HBeAg positive, HBV DNA load of ≥10^5^copies/ml; (2) the history of HBV infection ≥6 months; (3) the time of abnormal ALT ≥6 months; (4) the good compliance of patients. The excluded standards were as follows: (1) patients with antibodies against HIV or other forms of chronic liver disease; (2) patients with long term of drinking history; (3) patients complicated with fatty liver, the criteria of diagnosis of fatty liver by trans-abdominal ultrasonography: increased echogenicity, posterior attenuation and loss of intra-hepatic architectural details; (4) patients whose ALT were normal; (5) patients who were pregnant or in lactating; (6) patients with signs of variceal bleeding, ascites, encephalopathy. The study was approved by the Human Ethics Committee of The Third Affiliated Hospital, Sun Yat-sen University, Guangzhou, China. Informed written consent was obtained from each patient in the study.

### Study Design

All patients were treated with ADV, ETV, LAM or LDT, respectively. The standard of stopping therapy recommended by the 2005 APASL was as follows: anti-HBe seroconversion, HBV DNA<10^3^copies/ml (detection on 2 occasions at least 6 months apart). According to the different time period of the prolonged consolidation therapy with NUCs, patients were divided into 2 groups: group A and group B. Group A included 40 patients without the prolonged consolidation therapy. Group B was then categorized into B1, B2 and B3, depending on the time period of the prolonged consolidation therapy. Group B1 included 54 patients with 7 (range 3–11) months of the prolonged consolidation therapy. Group B2 included 23 patients with 17 (range 13–20) months of the prolonged consolidation therapy. Group B3 included 19 patients with 28 (range 25–34) months of the prolonged consolidation therapy. Sera of all patients were collected at baseline, and at 1, 2, 3, 4, 5, 6, 9, 12, 18, 24, 30, 36, 42, 48 months after off-therapy, respectively. HBV DNA, HBsAg, HBeAg, anti-HBe, ALT were analyzed, respectively. The standard of virological relapse was serum HBV DNA to >10^3^copies/ml.

### Laboratory Tests

Liver biochemistry was assayed by routine automated analysis system (Beckman Coulter, Fullerton, CA). HBV serological markers, including HBsAg, anti-HBs, HBeAg and anti-HBe were assayed by a chemiluminescent micro particle enzyme immunoassay (Abbott, Chicago, IL). The detection of serum HBV DNA level was previously described in detail [11].

### Statistical Analysis

Data were expressed as mean±SD or median. The Kaplan-Meier method was used to calculate the cumulative rate of relapse. Log-rank test was used to compare the cumulative rate of relapse among groups. The Cox’s proportional hazards regression model was adopted to determine the predictive factor for relapse, among various variables including age at off-therapy, gender, baseline HBV DNA level, baseline ALT level, baseline AST level, baseline TBIL level, baseline ALB level, time to virologic response, type of NUCs, the duration of the prolonged consolidation therapy and total treatment duration. Receiver operating characteristic curve (ROC curve) was used to calculate cut off value of the relevant predictive factor for relapse. All statistical analysis was performed using SPSS v16.00 statistical analysis software (SPSS Inc, Chicago, IL). Differences were considered statistically significant at a value of *P*<0.05.

## Results

### Patient Characteristics

One hundred and sixty-two HBeAg-positive CHB patients were recruited in the present study. After excluding 16 patients lost in follow-up period and 10 patients whose sera were not collected at baseline, 136 patients were eligible for this analysis. Sixty patients were treated with ADV, while 37 patients with ETV, 26 patients with LAM, 13 patients with LDT. Baseline characteristics of the 136 patients in this study were shown in Table 1.

**Table 1:** Baseline characteristics of the study population.

Factors	Group A	Group B1	Group B2	Group B3	F(X^2^/Z) value	*P* value
Gender(M/F)	28/12	46/8	18/5	13/6	(3.982)	0.226
Age (Year)	34.5±10.5	36.3±9.6	34.4±8.7	35.5±9.2	0.365	0.779
HBV DNA(log10 copies/ml)	5.8±1.3	5.7±1.3	6.2±1.2	6.0±0.8	1.436	0.242
ALB(g/L)	45.1±3.8	44.2±3.9	44.7±3.7	42.3±6.1	1.948	0.125
ALT (U/L)	93(66–217)	75(56–134)	75(55–186)	128(74–448)	(/4.164)	0.244
AST(U/L)	73(43–140)	78(56–147)	108(56–1725)	96(70–209)	(/3.670)	0.299
TBIL(µmol/L)	15(10–19)	15(11–24)	15(11–21)	15(10–25)	(/1.518)	0.678
Period of prolonged consolidation therapy (month)	0(0–0)	7(3–11)	17(13–20)	28(25–34)		
Time to HBeAg seroconversion (month)	28(14–40)	30(22–48)	34(30–50)	32(21–55)		
Time to undetectable HBV DNA (month)	17(14–27)	18(19–33)	25(22–43)	22(19–39)		
Total treatment duration (month)	35.8±5.0	40.1±6.4	45.9±7.3	59.3±17.3		
HBV genotype	All C					

### Cumulative Rate of Relapse

**Figure 1:**
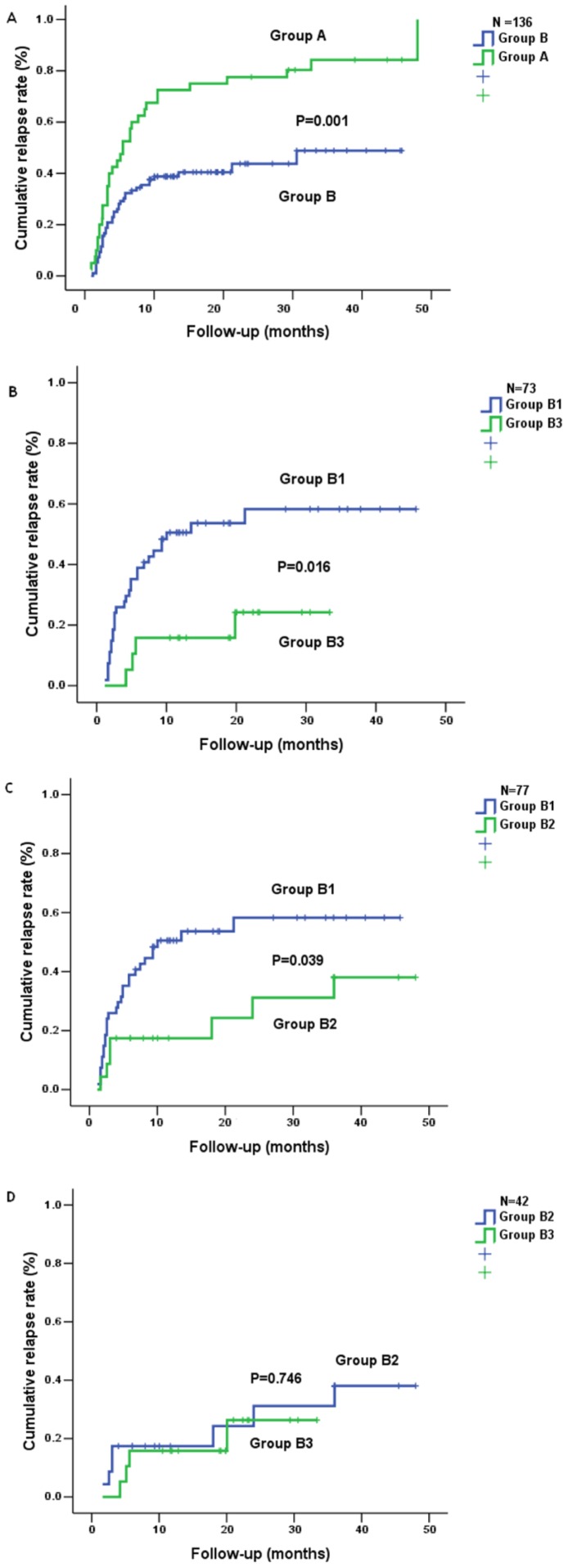
Cumulative rate of relapse in 136 CHB patients. Group A, 40 patients without the prolonged consolidation therapy. Group B, 96 patients with the different time period of the prolonged consolidation therapy. Group B1, 54 patients with 7 (range 3–11) months of the prolonged consolidation therapy. Group B2, 23 patients with 17 (range 13–20) months of the prolonged consolidation therapy. Group B3, 19 patients with 28 (range 25–34) months of the prolonged consolidation therapy. Fig. 1A showed the difference in the cumulative rate of relapse between group A and group B (*P*<0.05). Fig. 1B–D showed the difference in the cumulative rate of relapse among group B1, B2 and B3.

After off-therapy, the cumulative rates of relapse at 1, 2, 3, 4, 5, 6, 9, 12, 18, 24, 30, 36, 48 months were calculated, respectively. As shown in Fig. 1A, 52.5% patients recurred at 6 months in group A and only 29.2% patients at 6 months in group B. The cumulative rates of relapse in group A and group B at 48 months increased to 82.5%, 41.7%, respectively. Furthermore, the difference in the cumulative rate of relapse between group A and group B was statistically significant (Fig. 1A).

Subsequently, we compared the cumulative rates of relapse among group B1, group B2 and group B3. As shown in Fig. 1B–D, the cumulative rates of relapse in group B3 or group B2 were statistically lower than that in group B1, respectively. Although the difference in the cumulative rate of relapse between group B3 and B2 was not statistically significant, the cumulative rate of relapse in group B3 was lower than that in group B2.

### Relevant Factors for HBV Recurrence

Cox’s regression analysis revealed that HBV recurrence after off-therapy was associated with age at off-therapy (RR = 1.04; 95% CI, 1.021∼1.069; *P* = 0.000), baseline ALT level (RR = 0.999; 95% CI, 0.997∼1.000; *P* = 0.016) and the different time period of the prolonged consolidation treatment (RR = 0.974; 95% CI, 0.951∼0.998; *P* = 0.031). According to regression coefficients, the younger the patient was, or the higher the baseline ALT level was, or the longer the time period of the prolonged consolidation therapy was, the lower the rate of relapse was. The age was the most significant among the three relevant factors because the *Wald* of age was the highest. The time period of the prolonged consolidation therapy was the least significant among the three relevant factors because the *Wald* of the different time period of the prolonged consolidation therapy was the lowest (Table 2).

**Table 2:** Associated-factors of HBV recurrence by Cox’s hazards model.

Factor	*B*	*Wald*	*P*	*RR*	*RR 95% CI*
Age at off-therapy	0.044	13.462	0.000	1.045	(1.021, 1.069)
ALT at baseline	−0.001	5.818	0.016	0.999	(0.997, 1.000)
Different period of prolonged consolidation	−0.027	4.672	0.031	0.974	(0.951, 0.998)

Note: B, regression coefficient; Wald, *χ2* value; RR, risk ratio; CI, confidence interval.

Subsequently, ROC curves of the three relevant predictive factors were drawn and cut off values of them were calculated, respectively. Cut off value of age at off-therapy, ALT level at baseline, and the time period of the prolonged consolidation therapy, for predicting HBV recurrence, was 37 years old, 80 U/L, 11 months, respectively.

**Figure 2:**
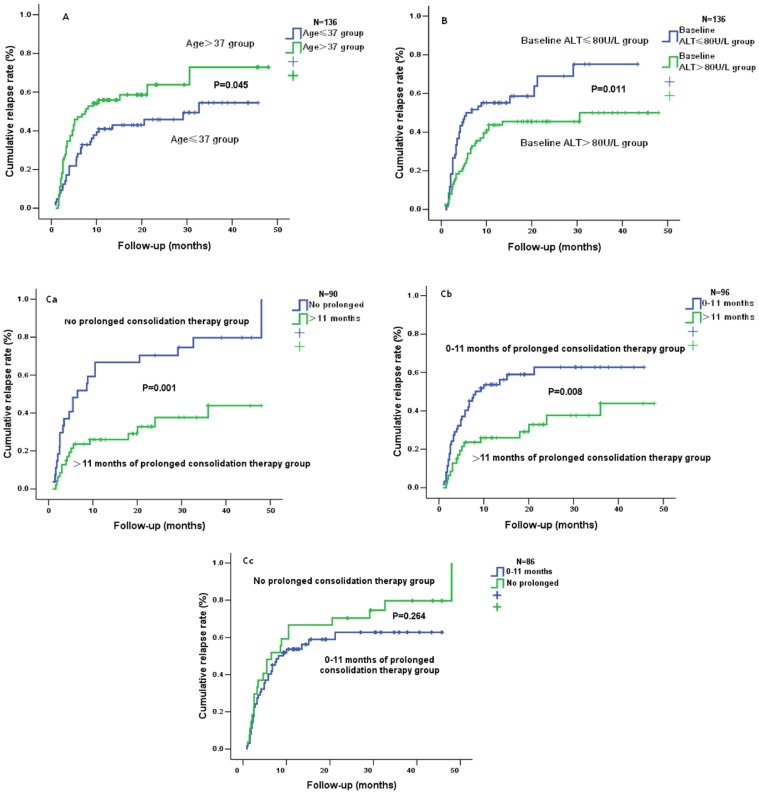
The difference in the cumulative rate of relapse between age ≤37 and age >37, ALT>80 U/L and ALT ≤80 U/L, the time period of the prolonged consolidation therapy>11 months and that ≤11 months. Fig. 2A showed the difference in the cumulative rate of relapse between age≤37 and age>37 (*P*<0.05). Fig. 2B showed the difference in the cumulative rate of relapse between ALT>80 U/L and ALT≤80 U/L (*P*<0.05). Fig. 2C showed the difference between the time period of the prolonged consolidation therapy>11 months and that ≤11 months (*P*<0.05).

Then we measured the differences in the cumulative rates of relapse between age≤37 and age>37, ALT>80 U/L and ALT≤80 U/L, the time period of the prolonged consolidation therapy>11 months and that ≤11 months, respectively. The cumulative rate of relapse in patient ≤37 years old at off-therapy (45.3%) was statistically lower than that in patient >37 at off-therapy (62.5%) (Fig. 2A). The cumulative rate of relapse in patient who had ALT >80 U/L at baseline (44.7%) was statistically lower than that in patient who had ALT ≤80 U/L at baseline (66.7%) (Fig. 2B). The cumulative rate of relapse in patient with >11 months of the prolonged consolidation therapy (26.0%) was significantly lower than that in patient with ≤11 months (58.7%) or that in patient without the prolonged consolidation therapy (85.0%) (Fig. 2Ca–b). Furthermore, the difference among them was statistically significant. However, the difference in the cumulative rate of relapse was not statistically significant between patient with 0–11 months of the prolonged consolidation therapy and patient without the prolonged consolidation therapy (Fig. 2Cc). These results further demonstrated the significance of cut off value.

## Discussion

In the present study, we found that the relapse rate of HBV after off-therapy gradually decreased with the increase of the time period of the prolonged consolidation therapy in HBeAg positive CHB patients. Age at off-therapy, baseline ALT level and the time period of the prolonged consolidation therapy were associated with recurrence of HBV after NUCs were discontinued. If patient was ≤37 years old at off-therapy, or ALT at baseline was >80 U/L, or the time period of the prolonged consolidation therapy was >11 months, the cumulative rate of relapse would significantly decrease after off-therapy.

For patients with HBV infection, it is best to eradicate HBV in hepatocyte, but there is no drug to eradicate HBV in hepatocyte worldwide. NUCs are widely used to treat CHB because they can suppress the replication of HBV and decrease the load of HBV DNA. However, patient with CHB often recurs because the load of HBV DNA rapidly rises after off-therapy [12]. It is demonstrated that high viral load (≥10^3^ copies/ml) is related to the progression of liver disease [13]–[14]. As a result, more attention is paid to the duration and the stopping point of NUCs therapy. APASL guidelines [6]–[7], AASLD guidelines [8] and EASL guidelines [1] recommend different duration and the stopping point of NUCs therapy for treating CHB with NUCs. In the present study, even if HBeAg positive CHB patient met the standard of stopping therapy recommended by the 2012 EASL guidelines [1], the cumulative rate of relapse was still high (Fig. 1). Cut-off value of the time period of the prolonged consolidation therapy also revealed that the cumulative rate of relapse in patient with >11 months of the prolonged consolidation therapy was significantly lower than that in patient with ≤11 months of the prolonged consolidation therapy (Fig. 2b)**.** Taken together, we believe that 12 months of the prolonged consolidation therapy after HBeAg seroconversion were imperfect for HBeAg positive CHB patients.

Covalently closed circular DNA (cccDNA), which functions as the template for the transcription of viral gene, is required for the maintenance of HBV infection [15]. Furthermore, it is difficult to eradicate cccDNA in hepatocyte [16]. Recently, Werle et al [17] demonstrates that long-term ADV therapy can significantly decrease cccDNA level in hepatocyte. The mechanisms they propose are as follows: the replication of HBV is suppressed by NUCs. The decrease of HBV replication results in the reduction of nucleotide chain. The reduction of nucleotide chain can not be converted into enough cccDNA to supplement the consumption of cccDNA. As a result, cccDNA level gradually lessens. In the present study, we found that the cumulative rate of relapse in patient with 28 (range 25–34) months of the prolonged consolidation therapy was the lowest in 136 patients. This might be associated with the decrease of cccDNA level. However, there were still 21.1% patients who recurred after 28 (range 25–34) months of the prolonged consolidation therapy in the present study. So we think that the consolidation therapy after HBeAg seroconversion should be further prolonged. Unfortunately, long-term NUCs therapy can result in HBV-resistance. All problems above are difficult to resolve.

The sustained durability in patient whose age was ≤37 was higher in the present study. Chien et al [18] demonstrates that patient ≤36 has a high sustained response. Song et al [19] demonstrates that patient ≤40 is significantly associated with the sustained response. Chu et al [20] reports that the incidence of hepatitis relapse is increased in patient who develops HBeAg seroconversion after age 40. Recently, it is reported that the difference in durability based on age can be associated with immune activity [21]. All these results demonstrated that it was more likely for younger patient to maintain viral suppression and high rate of durability after off-therapy. So we think that the consolidation therapy should be further prolonged for older patient to lessen the rate of relapse.

ALT level at baseline is an important predictive factor for the efficacy of antiviral therapy. Yan et al [22] approves that long-term efficacy of LAM is improved if the baseline ALT is more than 220 U/L. In the present study, we found that ALT level at baseline was associated with HBV recurrence after off-therapy and the cumulative rate of relapse was decreased if the baseline ALT was more than 80 U/L. Our results further demonstrated the role of ALT level in antiviral therapy.

It is reported that genotype B is related with spontaneous HBeAg seroconversion, and that genotype B patient has a significantly higher rate of spontaneous HBeAg seroconversion than genotype C patient [23]. It is also reported that genotype C patient is more HBeAg positive than genotype B patient [24]. Furthermore, genotype C is associated with more severe liver disease than genotype B [25]. So genotype C patient was recruited in the present study. Our results might be valuable for genotype C patient. Subsequently, in order to verify whether our results could also be meaningful for other genotype patients, we will investigate the relapse rate and the associated-factor of recurrence after stopping NUCs therapy with the different time period of the prolonged consolidation therapy in other genotype patients.

In sum, we have demonstrated that the APASL recommendations for stopping anti-viral therapy in HBeAg-positive CHB patients are inadequate. We, therefore, believe that the consolidation therapy with NUCs after HBeAg seroconversion should be further prolonged and the standard of stopping therapy should be further evaluated.

### Conclusion

Taken together, in the present study, we investigated the relapse rate and the associated-factor of recurrence after stopping NUCs therapy with the different time period of the prolonged consolidation therapy in HBeAg positive CHB patients. We revealed that the cumulative rate of relapse in patient with the prolonged consolidation therapy was statistically lower than that in patient without the prolonged consolidation therapy. Age at off-therapy, baseline ALT level and the different time period of the prolonged consolidation therapy were associated with HBV recurrence after off-therapy. Our results demonstrated that the consolidation therapy with NUCs after HBeAg seroconversion should be further prolonged, and that age at off-therapy, ALT at baseline and the time period of the prolonged consolidation therapy could provide information to direct anti-viral therapy.

## References

[1] European Association for the Study of the Liver (2012) EASL clinical practice guidelines: Management of chronic hepatitis B virus infection. J Hepatol 57: 167–185. 22436845 10.1016/j.jhep.2012.02.010

[2] Ganem D, Prince AM (2004) Hepatitis B virus infection–natural history and clinical consequences. N Engl J Med 350: 1118–1129. 15014185 10.1056/NEJMra031087

[3] Lok AS, McMahon BJ (2007) Chronic hepatitis B. Hepatology. 45: 507–539. 10.1002/hep.21513 17256718

[4] Hoofnagle JH, Doo E, Liang TJ, Fleischer R, Lok AS (2007) Management of hepatitis B: summary of a clinical research workshop. Hepatology 45: 1056–1075. 17393513 10.1002/hep.21627

[5] Maini MK, Boni C, Lee CK, Larrubia JR, Reignat S, et al (2000) The role of virus-specific CD8(+) cells in liver damage and viral control during persistent hepatitis B virus infection. J Exp Med 191: 1269–1280. 10770795 10.1084/jem.191.8.1269 PMC2193131

[6] Liaw YF, Leung N, Guan R, Lau GK, Merican I, et al (2005) Asian-Pacific consensus statement on the management of chronic hepatitis B: a 2005 update. Liver Int 25: 472–489. 15910483 10.1111/j.1478-3231.2005.01134.x

[7] Liaw YF, Leung N, Kao JH, Piratvisuth T, Gane E, et al (2008) Asian-Pacific consensus statement on the management of chronic hepatitis B: a 2008 update. Hepatol Int 2: 263–283. 19669255 10.1007/s12072-008-9080-3 PMC2716890

[8] Lok AS, McMahon BJ (2009) Chronic hepatitis B: update 2009. Hepatology 50: 661–662. 19714720 10.1002/hep.23190

[9] Reijnders JG, Perquin MJ, Zhang N, Hansen BE, Janssen HL (2010) Nucleos(t)ide analogues only induce temporary hepatitis B e antigen seroconversion in most patients with chronic hepatitis B. Gastroenterology. 139: 491–498. 10.1053/j.gastro.2010.03.059 20381492

[10] Xu XW, Lu MH, Tan DM (2005) Association between tumour necrosis factor gene polymorphisms and the clinical types of patients with chronic hepatitis B virus infection. Clin Microbiol Infect 11: 52–56. 15649304 10.1111/j.1469-0691.2004.01029.x

[11] Ke WM, Xie SB, Li XJ, Zhang SQ, Lai J, et al (2011) There were no differences in serum HBV DNA level between HBeAg-positive and HBeAg-negative chronic hepatitis B with same liver histological necroinflammation grade but differences among grades 1, 2, 3 and 4 apportioned by the same hepatic parenchyma cell volume. J Viral Hepat 18: 637–645. 21794025 10.1111/j.1365-2893.2011.01444.x

[12] van Nunen AB, Hansen BE, Suh DJ, Lohr HF, Chemello L, et al (2003) Durability of HBeAg seroconversion following antiviral therapy for chronic hepatitis B: relation to type of therapy and pretreatment serum hepatitis B virus DNA and alanine aminotransferase. Gut 52: 420–424. 12584227 10.1136/gut.52.3.420 PMC1773575

[13] Chen CJ, Yang HI, Su J, Jen CL, You SL, et al (2006) Risk of hepatocellular carcinoma across a biological gradient of serum hepatitis B virus DNA level. JAMA 295: 65–73. 16391218 10.1001/jama.295.1.65

[14] Iloeje UH, Yang HI, Su J, Jen CL, You SL, et al (2006) Predicting cirrhosis risk based on the level of circulating hepatitis B viral load. Gastroenterology 130: 678–686. 16530509 10.1053/j.gastro.2005.11.016

[15] Dandri M, Petersen J (2005) Hepatitis B virus cccDNA clearance: killing for curing? Hepatology 42: 1453–1455. 16317676 10.1002/hep.20976

[16] Glebe D (2007) Recent advances in hepatitis B virus research: a German point of view. World J Gastroenterol 13: 8–13. 17206750 10.3748/wjg.v13.i1.8 PMC4065879

[17] Werle-Lapostolle B, Bowden S, Locarnini S, Wursthorn K, Petersen J, et al (2004) Persistence of cccDNA during the natural history of chronic hepatitis B and decline during adefovir dipivoxil therapy. Gastroenterology 126: 1750–1758. 15188170 10.1053/j.gastro.2004.03.018

[18] Chien RN, Yeh CT, Tsai SL, Chu CM, Liaw YF (2003) Determinants for sustained HBeAg response to lamivudine therapy. Hepatology 38: 1267–1273. 14578866 10.1053/jhep.2003.50458

[19] Song MJ, Song DS, Kim HY, Yoo SH, Bae SH, et al (2012) Durability of viral response after off-treatment in HBeAg positive chronic hepatitis B. World J Gastroenterol. 18: 6277–6283. 10.3748/wjg.v18.i43.6277 PMC3501777 23180949

[20] Chu CM, Liaw YF (2007) Predictive factors for reactivation of hepatitis B following hepatitis B e antigen seroconversion in chronic hepatitis B. Gastroenterology. 133: 1458–1465. 10.1053/j.gastro.2007.08.039 17935720

[21] Lee CM, Ong GY, Lu SN, Wang JH, Liao CA, et al (2002) Durability of lamivudine-induced HBeAg seroconversion for chronic hepatitis B patients with acute exacerbation. J Hepatol 37: 669–674. 12399235 10.1016/s0168-8278(02)00267-2

[22] Yan J, Xie W, Wang Q, Li Y, Feng X, et al (2011) The optimal threshold: Baseline serum hepatitis B virus DNA and alanine transaminase levels can predict the 2-Year on-treatment virological response to lamivudine. Hepat Mon 11: 358–363. 22087161 PMC3212783

[23] Kao JH, Chen PJ, Lai MY, Chen DS (2004) Hepatitis B virus genotypes and spontaneous hepatitis B e antigen seroconversion in Taiwanese hepatitis B carriers. J Med Virol 72: 363–369. 14748059 10.1002/jmv.10534

[24] Orito E, Ichida T, Sakugawa H, Sata M, Horiike N, et al (2001) Geographic distribution of hepatitis B virus (HBV) genotype in patients with chronic HBV infection in Japan. Hepatology 34: 590–594. 11526547 10.1053/jhep.2001.27221

[25] Kao JH, Chen PJ, Lai MY, Chen DS (2000) Hepatitis B genotypes correlate with clinical outcomes in patients with chronic hepatitis B. Gastroenterology. 118: 554–559. 10.1016/s0016-5085(00)70261-7 10702206

